# Infants as Social Magnets: The Influence of Births on Social Interactions in Redfronted Lemurs (*Eulemur rufifrons*)

**DOI:** 10.1002/ajp.70067

**Published:** 2025-08-18

**Authors:** Amrei Pfaff, Claudia Fichtel, Peter M. Kappeler

**Affiliations:** ^1^ Behavioral Ecology and Sociobiology Unit, German Primate Center Leibniz Institute for Primate Research Göttingen Germany; ^2^ Department of Sociobiology/Anthropology, Johann‐Friedrich‐Blumenbach Institute of Zoology and Anthropology University of Göttingen Göttingen Germany

**Keywords:** allomaternal care, infant handling, parturition, social structure, twins

## Abstract

Infant survival is an important component of parental fitness in iteroparous species with slow life histories. From the infant's perspective, survival can be more or less directly influenced by the social environment, with group members potentially representing either a threat or a buffer against external stressors. Therefore, studying social relationship patterns during early development may provide insights into the effect of social factors on infant survival. To understand how group members interact with infants, and whether social relationships change due to the presence of infants, we conducted focal behavioral observations on four groups of wild redfronted lemurs (*Eulemur rufifrons*) during the birth season. Infant handling consisted mostly of grooming, while aggressive infant handling behaviors and allomaternal care occurred very rarely. Infants were groomed by individuals of all age‐sex classes at similar rates except for a trend of higher infant handling rates in juvenile females. After giving birth, mothers received more approaches and were closer in proximity to other group members than before birth, but there were no changes in grooming rates of mothers and other group members. Mothers also initiated more aggressive interactions towards other group members after giving birth. Therefore, other redfronted lemurs were clearly attracted to infants, which caused changes in affinitive relationships of mothers. At the same time, the increase in maternal aggression indicates that group members also represent some threat to infants. Our study provides a starting point for future studies, exploring how these early infant handling interactions and the mother's relationships impact an infant's subsequent survival, development and future relationships.

## Summary

- Mothers receive more approaches and are closer in proximity after giving birth, but affinitive interaction rates between nonmothers do not change.
- Mothers become more aggressive after giving birth.
- Male redfronted lemurs display similar infant handling rates as females.

## Graphical abstract

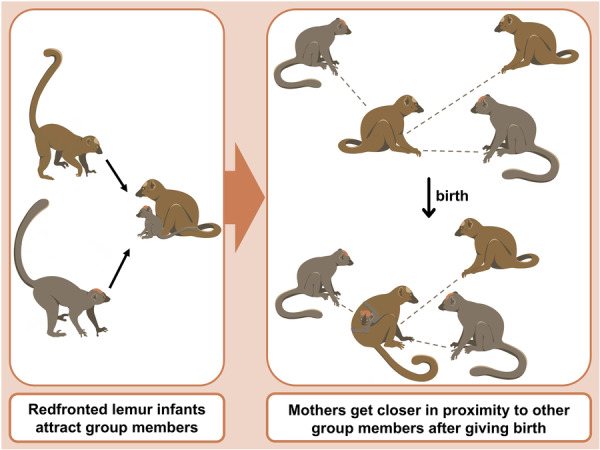

## 1 Introduction

The period following births is very critical for mammalian infants, as newborn offspring often face a heightened mortality rate (Berrut et al. 2016) due to both environmental and social risks (Kerhoas et al. 2014). In this regard, how other group members interact with an infant can be important for protection against certain stressors, including predation (Liu et al. 2018), but certain interactions can also represent a risk to the infant (Hrdy and Hausfater 1984; Kleindorfer and Wasser 2004). In addition to the direct influence of an infant's social interactions, infant survival can be influenced by a mother's social relationships as well (Silk et al. 2003). Mothers with stronger social bonds may enjoy increased infant survival and faster infant development (Cheney et al. 2016; Schneider‐Crease et al. 2022; Silk et al. 2003). As infant survival in primates and other mammals with slow life histories strongly impacts the reproductive success of the infant's parents, this period is not only critical for infants, but also their parents and other close kin. To protect their infants from ecological and social threats, mothers may change their social behavior towards other group members, especially in species where infanticide is common (Maestripieri 1994a). At the same time, the interest in infants of other group members can affect how they interact with mothers and potentially even relationships with non‐mothers (i.e., any individual without dependent offspring; Altmann 1980; Wei et al. 2013).

How individuals other than the mother interact with infants is described by the term infant handling (Maestripieri 1994a). Infant handling includes a wide range of different behaviors, which can be beneficial (e.g., carrying, allonursing), affiliative/neutral (e.g., touching, sniffing) or negative (e.g., pulling, aggression) for the infant (Dunayer and Berman 2018; Schino et al. 2003). Thereby, infant handling behaviors include, but are not restricted to, allomaternal care behaviors that provide a benefit for the infant (Clutton‐Brock 1991). There is some variation across previous studies in whether and how certain behaviors were included as aspects of allomaternal care; in particular, grooming is sometimes included as an allomaternal care behavior (e.g., Patel 2007; Tecot and Baden 2018). However, other researchers consider grooming to be distinct from allomaternal care, as it is not as necessary for infant survival as infant care behaviors such as lactation or carrying (e.g., Dunayer and Berman 2018; Hrdy 1976; Tecot et al. 2013). While attraction towards infants has been interpreted as a universality across primates, the expressed behaviors and amount of infant handling vary greatly among species (Dunayer and Berman 2018; Maestripieri 1994a). Infant handling rates do not depend solely on natal attraction towards infants but also maternal tolerance (Dunayer and Berman 2018). Therefore, interspecies variation in infant handling rates has been attributed to differences in social structure and maternal style (Maestripieri 1994a). As females in species with strong female‐female competition often direct more negative infant handling towards other females' infants, mothers are less tolerant of infant handling, resulting in lower infant handling rates (Chism 2000; Maestripieri 1994a).

Apart from a general variation among species, infant handling rates can also vary greatly between individual handlers and infants (Dunayer and Berman 2018; Nicolson 1987). In many species, there are clear differences between male and female handlers, with females handling infants more than males (e.g., Brent et al. 2008; Förster and Cords 2005; Silk 1999). Nonetheless, in some species, males do express similar or higher infant handling rates (e.g., common marmoset, *Callithrix jacchus*: Yamamoto and O.Box 1997; Barbary macaques, *Macaca sylvanus*: Paul 1999; blue‐eyed black lemur, *Eulemur flavifrons*: Volampeno et al. 2011). Species also vary in the most common age class of handlers (Dunayer and Berman 2018), with young, nulliparous females having the highest infant handling rates in some species, while in other species, adult, parous females handle infants more (reviewed in Dunayer and Berman 2018). In addition, rare situations, such as twin births in monotocous species, may also cause increases in infant handling rates (Bennett 1988; Chapman and Chapman 1986; Dielentheis et al. 1991).

Since infants attract the attention of other group members, mothers are also often described as attractive because they receive more affiliative (i.e., socio‐positive behaviors) and affinitive (i.e., proximity seeking) behaviors (Matsumura 1997; Seyfarth 1976). Mothers are frequently approached more often (Martel et al. 1994; Slater et al. 2007; Wei et al. 2013) and receive more grooming (e.g., Jiang et al. 2019; Manson 1999; Struhsaker 1971). This increase in grooming has been explained within the framework of biological markets, where mothers exchange access to their infants against other commodities, such as grooming (Henzi and Barrett 2002). At the same time, mothers adapt their own social behavior, as they focus on their infant (Altmann 1980). They often decrease the amount of time they groom others, which has been described in several cercopithecine monkeys (e.g., moor macaques *Macaca maurus*: Matsumura 1997; olive baboons *Papio anubis*: Frank and Silk 2009; Tibetan macaques *Macaca thibetana*: Jiang et al. 2019) as well as golden snub nosed‐monkeys (*Rhinopithecus roxellana*; Wei et al. 2013) and tufted capuchin monkeys (*Sapajus apella*; Tiddi et al. 2010), or are less likely to reciprocate grooming (Muroyama 1994). Therefore, grooming interactions with mothers are often more asymmetrical or completely unidirectional (Frank and Silk 2009; Gumert 2007).

While changes in maternal relationships have been studied in a variety of species, few studies have considered more general differences in social interactions in nonmother dyads (Rasmussen et al. 2003; Schino and Troisi 2001; Wei et al. 2013) or across the entire group (Díaz et al. 2023; Wikberg et al. 2015). Theoretically, if individuals interact more with mothers, there could be two potential outcomes: (I) Nonmother individuals may maintain their interaction rates with other nonmothers, which would result in an increase in their overall time spent being social. Alternatively, if there is a constraint on time (Lehmann et al. 2007), (II) they may maintain their total time spent being social, which would require a decrease in the interaction rates towards other nonmothers. So far, there exists limited evidence for both possibilities. Wikberg et al. (2015) found that overall grooming rates were higher in ursine colobus monkey (*Colobus vellerosus*) groups with higher proportions of mothers, suggesting that increases in grooming directed towards mothers are not constrained by maintenance of their overall time spent being social. However, approach rates of nonmother dyads decreased after the birth of infants in female golden snub‐nosed monkeys (Wei et al. 2013). Similarly, in cotton‐top tamarins (*Saguinus oedipus*) grooming network density decreased after the birth of infants, suggesting that fewer dyads were grooming each other (Díaz et al. 2023). As a consequence, the presence of infants may have more far‐reaching effects, for instance on social cohesion, which can further be influenced by changes in aggression rates.

The presence of infants can lead to an increase in aggression between non‐infant group members due to a variety of reasons (Hrdy 1976; Maestripieri 1992, 1994a). As mothers attempt to protect their newborn offspring, they often act more aggressively (Maestripieri 1992; Pereira and Weiss 1991). This aggression may be directed towards strange males or non‐fathers (Pereira and Weiss 1991), but can also be directed towards other group members who attempt to handle infants (Henzi and Barrett 2002; Maestripieri 1992; Troisi 1988). In some species, mothers associate with one or two males (Baniel et al. 2016; Nguyen et al. 2009; Palombit et al. 1997), who also use aggression to protect infants (Altmann 1980; Fruteau et al. 2010; Hrdy 1976). In addition, conflict between nonmothers can arise during competition over access to the mother and her infant (Cheney 1978; Seyfarth 1976). Finally, mothers may also be the recipient of aggression, especially from other females (Baniel et al. 2018), which has been assumed to occur mostly in species with strong female‐female competition (Maestripieri 1994a; Wasser 1983).

While many studies have examined changes in the social relationships of mothers, little is known about how these variations may affect the wider group dynamics. Some previous studies indicated the potential of group‐level effects (Díaz et al. 2023; Wikberg et al. 2015), but little is known about the changes in dyadic relationships which cause them. Moreover, most previous studies have been carried out in catarrhine primates (e.g., Altmann 1980; Bruce et al. 1988; Caselli et al. 2021), which typically live in relatively large groups (Kappeler and Heymann 1996). However, small changes in individual social relationships may have greater consequences in species that live in small groups, such as many lemurs.

This study thus focused on redfronted lemurs (*Eulemur rufifrons*), who live in small multimale‐multifemale groups. Their reproduction is highly seasonal, with births occurring between late September and October, and females of the same group typically giving birth only a few days apart (Ostner and Kappeler 1999), which may cause a relatively strong and abrupt change in social dynamics. So far, little is known about the changes in social interactions following births as well as the patterns of infant handling in redfronted lemurs. However, mothers have been described to separate themselves spatially from other group members (Overdorff 1998). In comparison to other *Eulemur* species, redfronted lemurs are not known to display allomaternal care (Tecot et al. 2013), but infants receive grooming from other group members (Barthold et al. 2009).

In this study, we examined patterns of infant handling and compared social relationships before and after the birth of infants, to determine the influence of births on social relationships. We hypothesized that births influence different dyadic social relationships in different ways. Based on previous studies mentioned above, we predicted that (I) mothers will be attractive to other group members and therefore (Ia) receive more grooming and (Ib) more approaches. As other group members focus their attention on new mothers, we expected them (II) to interact less with other nonmothers, in the form of grooming and approaches. In addition, we predicted (III) a decrease in spatial cohesion, because mothers may avoid other group members, which has been described previously (Overdorff 1998), or because of reduced interactions between nonmothers. Finally, we predicted (IV) an increase in aggression, as especially mothers may act aggressively towards other group members to prevent potentially harmful infant handling.

## 2 Methods

### 2.1 Ethical Note

The study adhered to the American Society of Primatologists (ASP) Principles for the Ethical Treatment of Nonhuman Primates and followed the ASP Code of Best Practices for Field Primatology. The study further adhered to the legal requirements of the country (Madagascar) in which the work was conducted and complied with the protocol approved by the Malagasy Ministry of the Environment, Water, and Forests (No. 293/23/MEDD/SG/DGGE/DAPRNE/SCBF.Re).

### 2.2 Study Site and Subjects

Redfronted lemurs live in small multimale‐multifemale groups with philopatric females (Overdorff et al. 1999; Prox et al. 2023). Females begin reproducing at 3.6 years on average and give birth to single offspring with an average interbirth interval of 1.2 years (Kappeler et al. 2022; Overdorff et al. 1999). Infants first start being off their mother after approximately 5 weeks and trying solid food at about 8 weeks of age. They start moving independently over large distances after approximately 12 weeks (Barthold et al. 2009) and are weaned after approximately 4.5 months (Kappeler and Pereira 2003). All infants are born with a natal coat resembling the adult male coloration of this sexually dichromatic species (Barthold et al. 2009). Aggression rates are typically very low (Seex et al. 2022), but female‐female competition can escalate into female evictions (Kappeler and Fichtel 2012; Prox et al. 2023). While redfronted lemurs do not express a clear dominance hierarchy, there is usually one central male who is dominant over the other males and has higher mating and reproductive success (Kappeler and Port 2008; Ostner and Kappeler 1999).

All data were collected at Kirindy Forest (44°39'E; 20°03'S), a dry‐deciduous forest in Madagascar between 10 September and 22 November 2023. We observed 31 individuals in four habituated groups, which ranged in size between 4 and 11 individuals (Table S1). In one group, shortly after the births of the first infants, one male disappeared and was therefore excluded from all analyses. While his disappearance might have caused slight changes in interaction rates between other group members, this is unlikely, as disappearances have overall little impact on social structure in this species (Pfaff et al. 2023), and he had only joined the group very recently and was thus not well integrated.

During the observation period, 11 females gave birth (Table S2). While most infants were singletons, three females gave birth to twins. In the first 25 years of this long‐term study, only 1 twin birth occurred among 201 singleton births (Kappeler et al. 2022). Two infants disappeared during the study period at approximately 1 month of age. As we were only able to collect data on 2 days while infant Sapbb was alive, we excluded this infant from all further analyses.

### 2.3 Data Collection

We performed 30 min focal animal observations, where we recorded continuous data on social interactions following an established ethogram (Pereira and Kappeler 1997) with the addition of infant handling behaviors. Infant handling was divided into beneficial behaviors, such as carrying and holding, affiliative/neutral behaviors, which included grooming, touching and inspecting, and negative behaviors, which included hitting, biting and pulling (Table 1). All group members were used as focal individuals with the exception of infants. While we did not include infants as focal individuals, we noted infant handling interactions when observing their mothers or the handler. As it was impossible to distinguish between the twins during the first weeks, they were treated as one individual when recording data. We further conducted proximity scan samples every 15 min, where we recorded the proximity of all other group members in relation to the focal individual, within the categories of inside a radius of 1, 3, 5, 10 m or more than 10 m. Due to their rarity of allomaternal care and aggressive infant handling, any occurrences not involving the focal individual that were noticed during observation time were noted as ad libitum observations.

**TABLE 1:** Ethogram of infant handling behaviors.

Behavior	Definition	Type
Inspecting	Bringing face within 10 cm of an infant and looking at it.	Duration
Grooming	Repeated stroking over an infant's fur using tooth comb or tongue. As usually very little allogrooming is directed at the ventrum (Barton 1985), infant grooming was also recorded when the mother's ventrum was groomed and it was impossible to distinguish whether the grooming was directed at the infant or the mother.	Duration
Holding	Resting with an infant clinging to their ventrum.	Duration
Carrying	Moving, feeding or foraging with an infant clinging to their ventrum.	Duration
Touching	Making contact with an infant using hands.	Frequency
Pulling	Grabbing and pulling an infant.	Frequency
Aggression	Hitting or biting an infant.	Frequency

Observations were carried out between 07:00–11:30 and 14:00–18:00 h, with one group being observed in the morning and afternoon each. Each individual was focaled not more than once per day, and the order of focal observations was pseudo‐randomized to ensure similar observation durations throughout the day. In total, approximately 174.9 h of data were collected (85.8 h pre‐birth and 89.1 h post‐birth), Observation times ranged between 21.7 and 57.8 h per group (9.6–31.5 h pre‐birth and 12.1–29.1 h post‐birth) and between 4.9 and 7.5 h per individual (2.0–4.0 h pre‐birth and 1.9–3.7 h post‐birth) with a mean of 6.2 ± 0.7 h (2.9 ± 0.6 h pre‐birth and 3.0 ± 0.5 h post‐birth).

### 2.4 Data Analyses

We first examined rates of different infant handling behaviors based on focal observation data and ad libitum observations. As infant behaviors occurred at very different frequencies and only infant grooming was observed frequently, we restricted our further analysis of calculated dyadic infant handling rates on infant grooming rates from focal observation data. To examine whether these dyadic infant handling rates were influenced by the age and sex of the handler or by the occurrence of twins, we fitted a Generalized Linear Mixed Model (GLMM; Baayen 2008) with beta error distribution and logit link function. Dyadic infant grooming rates (proportion of time) were used as the response. As fixed effects, we included handler age (adult/juvenile), handler sex (female/male) and an interaction between the two factors, as well as twinning (singleton/twins). To control for potential effects of infant availability and competition between handlers, we further included group size and the number of mothers per group, as well as an interaction between them. The identities of the infant, handler and group were included as random factors. We included all theoretically identifiable random slopes (Barr et al. 2013; Schielzeth and Forstmeier 2009), namely handler age and sex within infant and group. However, correlations between random intercept and slopes were removed, as the model did not converge initially. Variance Inflation Factors (VIF; Field 2005) values revealed collinearity between group size and number of mothers, with a VIF of 2.45. The model was not overdispersed (dispersion parameter = 1.11).

To examine whether changes in social relationships occurred due to the presence of newborn infants, we calculated interaction rates in an approximate 1‐month timeframe before and after the birth of infants. The before‐birth period ended at the latest as soon as the first infant was born in a group. However, as groups varied in the level of birth synchrony, our approach to determine the after‐birth timeframes varied between groups. For group J, where births were only a few days apart with only 2 days of data collection in between, we determined the second birth as the start of the after‐birth period and discarded the data collected in between the births from all analyses. In group alpha, we used two different after‐birth timeframes to determine interaction rates of different dyads, since there was a large difference in time (approximately 4 weeks) between the first and last births. To not exclude a large amount of data between the births, we used the first birth as the start of the after‐birth timetable of all nonmother‐nonmother dyads, as well as for the dyads of the mothers whose infants had been born at this date (Rbd, Gen). For the dyads which included mothers whose infants were born later (Red, Flo), the birth date of their infants was used as the start of the after‐birth period.

For every dyad, we calculated directional rates of grooming (proportion of time), approaches and aggressive behaviors (including hit, bite, body threat and chase) per hour. We further calculated dyadic proximity measures based on the scan samples using C scores (= composite proximity measure; Smuts 1985), where different proximity categories are weighted by corresponding factors. We used the formula:

$$C=P_{0-1}*1+P_{1-3}*1/4+P_{3-5}*1/8+P_{5-10}*1/15,$$

where *P*_A__−B_ is the proportion of scans a dyad spent within a radius between A and B meter. The corresponding weighting factors were determined by calculating the inverse of the mid‐point of each range, which was then multiplied by two, to ensure that C scores varied between zero and one (with zero suggesting that a dyad was never observed within a 10 m distance and one suggesting that a dyad was always within a 1 m distance).

To estimate whether the presence of infants affected grooming, approach and aggression rates, we fitted three GLMMs. The grooming rate model contained beta error distribution and logit link function, while the approach and aggression model had Poisson error structure and log link function (McCullagh and Nelder 1989). All models included period (before/after birth), giver‐type (mother/nonmother) and receiver‐type (mother/nonmother) as fixed effects. As we expected different changes for mothers and nonmothers and different dyad types, we included a three‐way‐interaction between period, giver type and receiver type, as well as all possible two‐way interactions. However, in the aggression model, the three‐way‐interaction would have resulted in complete separation. Therefore, we only included all two‐way‐interactions between period, giver‐type and receiver‐type in this model. We included group size as a control factor and the identities of the giver and receiver, the dyad, and the group as random factors. To control for variation in observation duration between dyads, we included it as a weight in the grooming model and as an offset term in the approach and aggression model (log‐transformed with base e; McCullagh and Nelder 1989).

The approach and aggression model included all theoretically identifiable random slopes, namely the interaction between period*giver‐type within receiver identity, the interaction period*receiver‐type within giver identity and the three‐way‐interaction of period*giver‐type*receiver‐type (approach model) or all two‐way interactions between period, giver‐type and receiver‐type (aggression model) within group identity. Due to convergence issues, the approach model did not contain any correlations between random intercepts and slopes and the aggression model contains correlations only within giver and receiver identity. The grooming model only contained the random slopes within giver and receiver type and no correlations. For further information on the model fitting process see the Supporting Information.

Collinearity was not an issue in all three models, as the maximum VIF was 1.68. The approach and aggression models were not overdispersed with corresponding dispersion parameters of 0.77 and 0.35. However, the grooming model did express overdispersion (dispersion parameter = 2.02), therefore, we corrected the model results for overdispersion by multiplying the standard errors with the square root of the dispersion parameters and then recalculating *p*‐values (Gelman and Hill 2007).

To estimate whether births lead to changes in proximity, we fitted a Bayesian Multimembership Model with beta error distribution and logit link function, to account for the proximity rates being determined by both members of a dyad (Martin et al. 2021). As fixed effects, we included period (before/after) and dyad type (mother‐mother/mother‐nonmother/nonmother‐nonmother), as well as an interaction between the two terms, as we expected different changes in different types of dyads. Additionally, the model included group size as a control factor. As random effects, we included the identity of both individuals (as a multimembership term), the dyad, and the group. All theoretically identifiable random slopes were included, namely dyad type within group identity and period within group and individual identity. In addition, all correlations between random intercepts and slopes were included.

## 3 Implementation

In all models, the quantitative predictors (namely group size and number of mothers) were z‐transformed to a mean of zero and a standard deviation of one to ease model convergence and the interpretability of model estimates (Schielzeth 2010). All factors were manually dummy‐coded and centered to a mean of zero before including them in the random effects structures. In the infant handling, grooming and proximity model, the responses were transformed to avoid values from being zero or one (Smithson and Verkuilen 2006). For all GLMMs, we compared the full models with null models (lacking handler sex, age and twinning for the infant handling model, and lacking period and its interactions for the grooming, approach and aggression model) to test the overall effect of the factors of interest and avoid “cryptic multiple testing” (Forstmeier and Schielzeth 2011). To test the effect of individual fixed effects, we compared the full model with reduced models lacking one fixed effect at a time. We utilized “likelihood ratio tests” (Dobson 2002) for these tests and the full‐null model comparison. We estimated model stability by dropping the random factor levels one at a time from the data and comparing resulting model estimates (Nieuwenhuis et al. 2012). The approach and aggression model expressed relatively good stability (Figures S1, S2), while the stability of the grooming and infant handling model was only moderate (Figures S3, S4) and should thus be interpreted with some caution.

We fitted all models in R (version 4.4.0; R Core Team 2024) using the functions glmmTMB of the package glmmTMB (version 1.1.9; Brooks et al. 2017), glmer of the package lme4 (version 1.1‐35.3; Bates et al. 2015), and brm of the package brms (version 2.21.0; Bürkner 2017). We determined VIFs using the function vif of the package car (version 3.1‐2; Fox and Weisberg 2019). We used the function simulate of the package lme4 and glmmTMB, to obtain confidence intervals of GLMM model estimates and fitted values using parametric bootstrapping (*N* = 1000).

The sample of the infant handling model included 60 dyadic rates of eight infants and 28 handlers across four groups. For the grooming, approach and aggression model, the sample included 428 observations based on 30 individuals (both giver and receiver) from four groups forming 214 dyads. The proximity model was based on 214 observations of 107 dyads from 30 individuals across four groups.

## 4 Results

### 4.1 Infant Handling

There were large differences in the relative occurrences of different types of infant handling behaviors. Allomaternal care, in the form of holding and carrying infants by other individuals, occurred only rarely, as it was only observed on seven occasions, consisting of four occurrences during focal observations and three ad lib. observations. The handler in these situations was either an adult male or an adult female, and the interaction lasted between approximately 30 s and 30 min. Aggression directed towards infants also occurred very rarely, as it was observed only once during focal observations, where a mother hit the infant of another female. In addition, in three ad lib. observations, aggression directed at infants was observed from adult males. Overall, affiliative or neutral infant handling behaviors were the most common, with a particularly high frequency of infant grooming. Inspecting infants occurred relatively regularly, with a total of 18 observations. Grooming was the most common infant handling interaction, with a total of 352 observed grooming interactions that lasted on average 22.5 ± 25.4 s.

**FIGURE 1:**
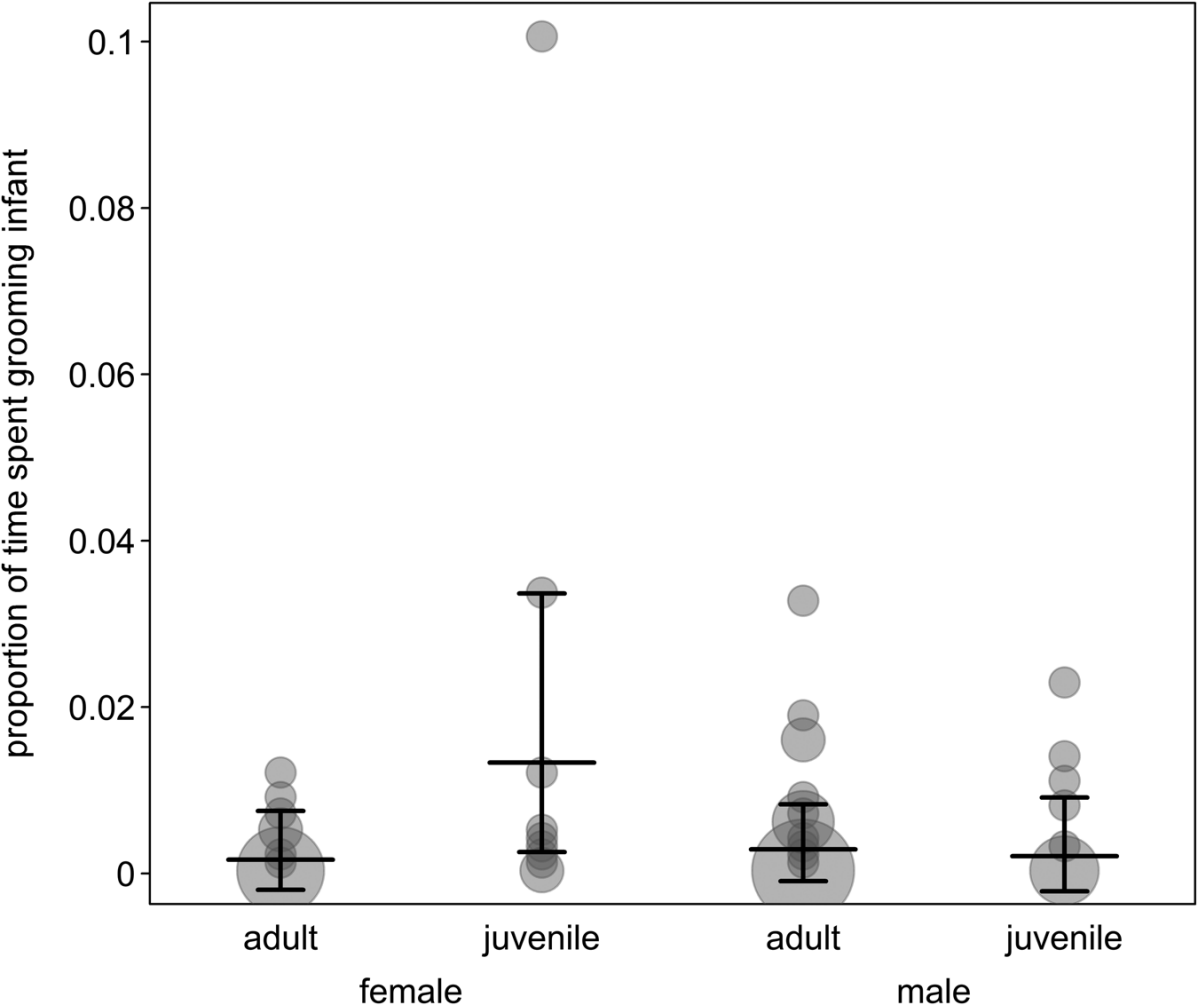
Infant handling rates depend on sex and age of the handler. Dots depict the proportion of time spent on infant grooming, with the area of the dots corresponding to the number of dyads (range: 1 to 11). Horizontal line segments and error bars depict the fitted model and its confidence limits for group size and number of mothers being at its average and twinning being centered.

To examine whether infant grooming rates varied between different age‐sex classes, we fitted a GLMM. The comparison between the full infant handling model and the null model revealed that the full model was not significantly better than the null model (*X* ^*2*^ = 7.49, *df* = 4, *p* = 0.11). Therefore, the following results should be treated with caution, as significant results may be due to cryptic multiple testing. The interaction between age and sex was significant (Table 2), suggesting that juvenile females groomed infants more than adult females, while no difference between the ages was present in males (Figure 1). In addition, there was no difference in infant grooming rates between adult females and males. However, the higher rates of infant grooming in juvenile females were strongly driven by one single individual. The occurrence of twins did not seem to affect infant handling, as infant grooming rates did not increase in the case of twinning in comparison to single infants (Table 2).

**TABLE 2:** Results of the infant handling model (including estimates, standard errors, confidence levels and significance tests).

Term	Estimate	SE	*CL*_lower_	*CL*_upper_	*X* ^*2*^	*df*	*p*
Intercept	−4.587	0.268	−5.098	−4.117			
Sex†	0.122	0.249	−0.373	0.632			
Age†	0.804	0.402	0.090	1.403			
Twinning†	−0.135	0.139	−0.378	0.128	1.011	1	0.315
Group_size‡	0.054	0.383	−0.574	0.761			
Number_Mothers§	−0.206	0.221	−0.596	0.180			
Sex:Age	−0.885	0.435	−1.620	−0.110	4.629	1	**0.031**
Group_size:Number_Mothers	0.135	0.209	−0.229	0.512	0.417	1	0.519

^†^ Dummy coded with female, adult and singleton being the reference level, respectively.

^‡^ z‐transformed; mean and sd of original group size were 9 and 1.496, respectively.

^§^ z‐transformed; mean and sd of original number of mothers were 3.033 and 1.235, respectively.

### 4.2 Changes in Social Relationships

We found no indication that grooming rates changed after the birth of infants, since the full grooming model was not significant compared to the null model (*X* ^*2*^ = 4.65, *df* = 4, *p* = 0.33). After accounting for overdispersion all grooming model estimates were also clearly nonsignificant (Table 3).

**TABLE 3:** Results of the grooming model (including estimates, standard errors, confidence levels and significance tests; corrected for overdispersion).

Term	Estimate	SE	*CL*_lower_	*CL*_upper_	*p*
Intercept	−6.101	0.599	−7.007	−5.281	
Period†	−1.027	0.853	−2.408	0.170	
Giver‐type‡	−0.147	0.662	−1.154	0.803	
Receiver‐type‡	−0.238	0.656	−1.222	0.707	
Group size§	−0.037	0.102	−0.198	0.102	0.715
Period:giver‐type	1.288	0.955	−0.027	2.792	
Period:receiver‐type	0.950	0.948	−0.409	2.476	
Giver‐type:receiver‐type	0.296	0.759	−0.787	1.398	
Period:giver‐type:receiver‐type	−1.417	1.060	−3.143	0.088	0.181

^†^ Dummy coded with before being the reference level.

^‡^ Dummy coded with mother being the reference level.

^§^ z‐transformed; mean and sd of original group size were 8.827 and 1.719, respectively.

**FIGURE 2:**
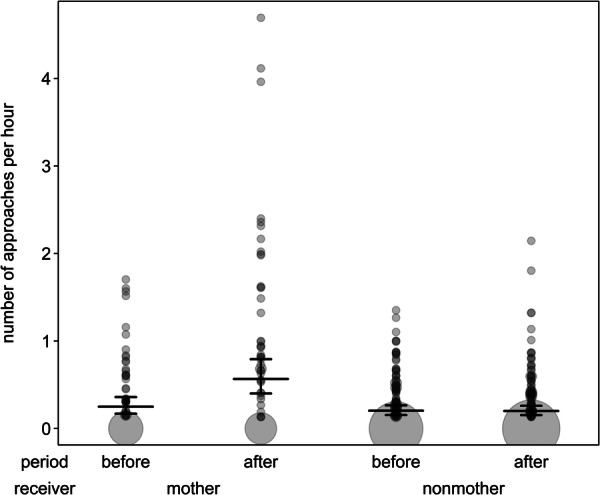
Number of approaches received by mothers and nonmothers before and after the birth of infants. Dots depict the number of approaches per hour, with the area of the dots corresponding to the number of dyads (range: 1 to 64). Horizontal line segments and error bars depict the fitted model and its confidence limits for group size being at its average and giver‐type being centered.

Regarding approaches, the full model was significantly better than the null model (*X* ^*2*^ = 12.46, *df* = 4, *p* = 0.01). However, the three‐way‐interaction between period, giver‐type and receiver‐type did not have a significant effect in the approach model (*X* ^*2*^ = 0.07, *df* = 1, *p* = 0.79). Therefore, we report here results from a reduced model lacking this three‐way‐interaction and the interaction between period and giver type and between giver‐type and receiver‐type, as these interactions were also nonsignificant (*X* ^*2*^ = 1.67, *df* = 1, *p* = 0.20; *X* ^*2*^ = 1.83, *df* = 1, *p* = 0.18). We found a significant effect of the interaction between period and receiver‐type (Table 4), which suggests that there was a stronger increase after infants were born in approaches received by mothers compared to nonmothers (Figure 2). In addition, nonmothers generally approached other individuals more than mothers, independent of receiver‐type and period, and approaches were in general less frequent in larger groups than smaller groups (Table 4).

**TABLE 4:** Results of the reduced approach model (including estimates, standard errors, confidence levels and significance tests).

Term	Estimate	SE	*CL*_lower_	*CL*_upper_	*X* ^*2*^	*df*	*p*
Intercept	−1.807	0.283	−2.426	−1.285			
Period†	0.819	0.232	0.151	1.102			
Giver‐type‡	0.583	0.216	0.446	1.236	5.209	1	**0.022**
Receiver‐type‡	−0.204	0.213	−0.587	0.237			
Group size§	−0.186	0.093	−0.361	0.014	3.856	1	**0.049**
Period:receiver‐type	−0.833	0.242	−1.293	−0.382	6.846	1	**0.001**

^†^ Dummy coded with before being the reference level.

^‡^ Dummy coded with mother being the reference level.

^§^ z‐transformed; mean and sd of original group size were 8.827 and 1.719, respectively.

**FIGURE 3:**
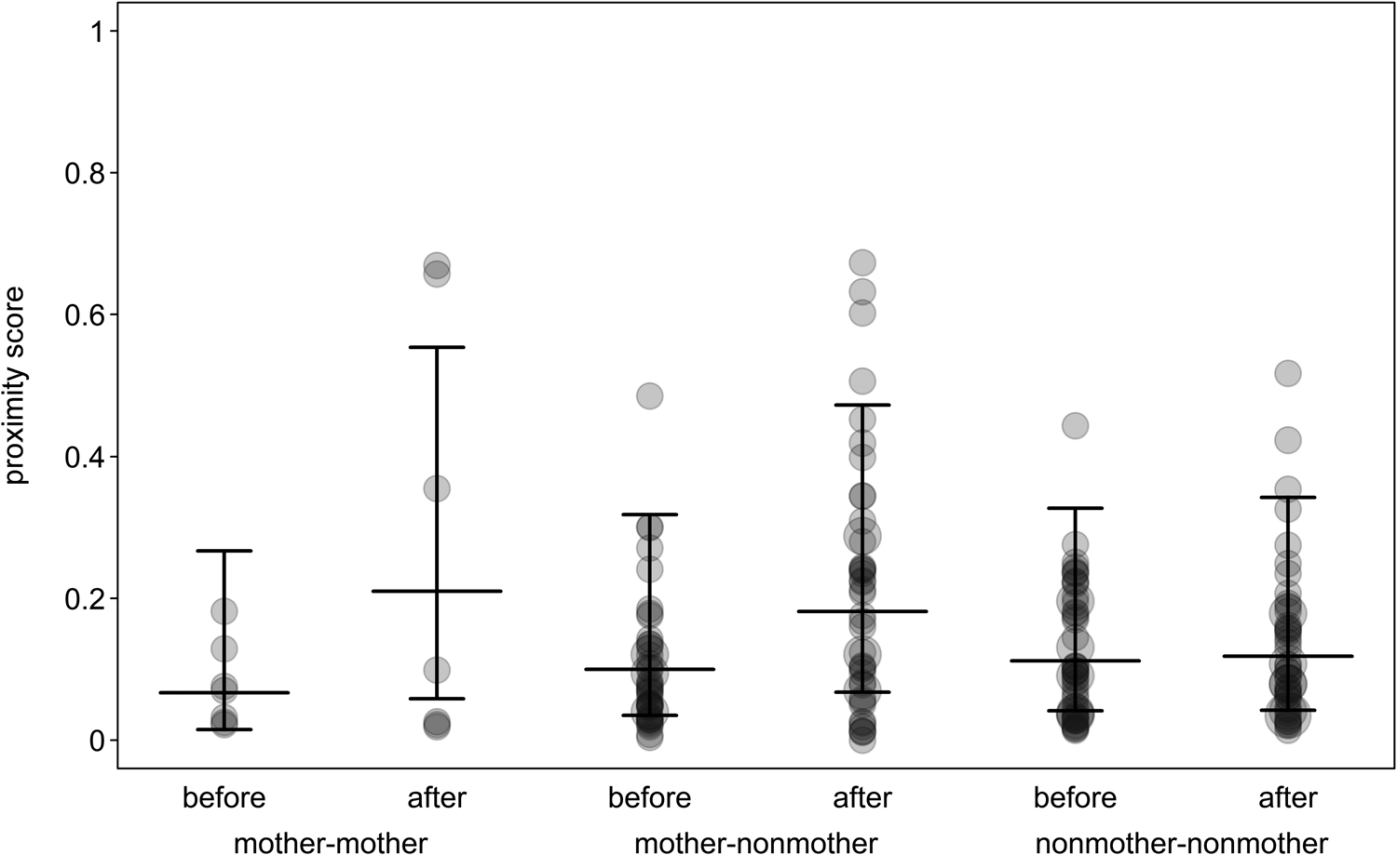
Proximity of different dyad types before and after the birth of infants. Dots depict the proximity scores, with the area of the dots corresponding to the number of dyads (range: 1 to 3). Horizontal line segments and error bars depict the fitted model and its credible intervals.

The proximity model revealed that there was a difference in the impact of births on mother‐mother and nonmother‐nonmother dyads, as the 95% credible interval of the interaction between period and dyad‐type‐nonmother‐nonmother did not include zero (Table 5). This suggests that mother‐mother dyads increased their proximity after births, while the proximity between nonmother‐nonmother dyads did not change (Figure 3). A similar difference was not found between mother‐mother and mother‐nonmother dyads (Table 5), suggesting that proximity also increased in mother‐nonmother dyads, although by a smaller amount.

**TABLE 5:** Results of the proximity model (including estimates, estimated errors and 95% credible intervals of model estimates).

Term	Estimate	Est. error	Lower‐95% CI	Upper‐95% CI
Intercept	−2.65	0.73	−4.17	−1.21
Period†	1.26	0.50	0.30	2.25
Dyad‐type.MO‐NM‡	0.52	0.68	−0.70	1.93
Dyad‐type.NM‐NM‡	0.59	0.71	−0.82	1.98
Group size§	−0.28	0.23	−0.82	0.11
Period:dyad‐type.MO‐NM	−0.67	0.43	−1.53	0.16
Period:dyad‐type.NM‐NM	−1.26	0.46	−2.19	−0.37

^†^ Dummy coded with before being the reference level.

^‡^ Dummy coded with mother‐mother (MO‐MO) being the reference level.

^§^ z‐transformed; mean and sd of original group size were 8.827 and 1.721, respectively.

**FIGURE 4:**
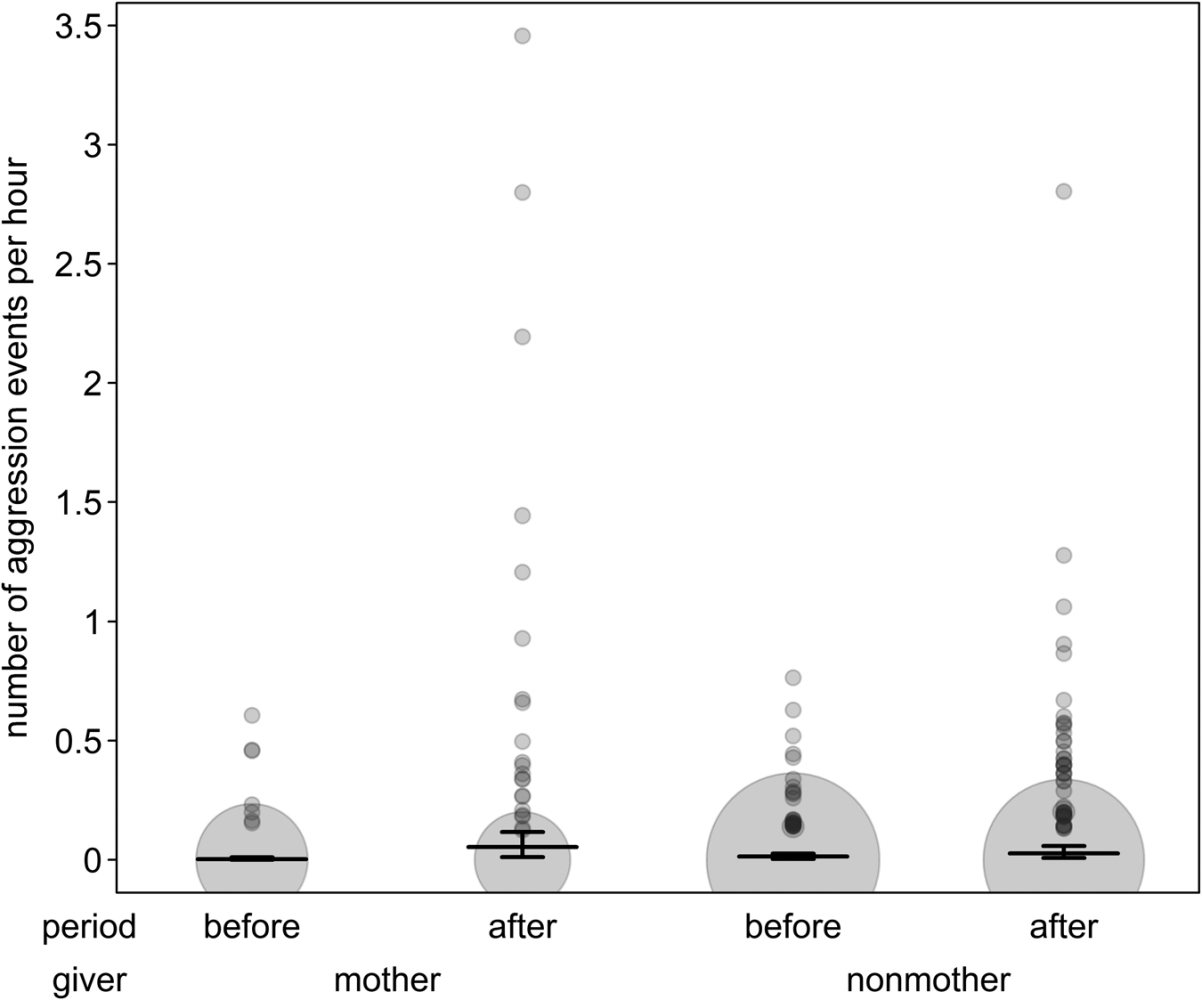
Number of aggression events given by mothers and nonmothers before and after birth of infants. Dots depict the number of aggression events per hour, with the area of the dots corresponding to the number of dyads (range: 1 to 127). Horizontal line segments and error bars depict the fitted model and its confidence limits for group size being at its average and receiver‐type being centered.

The aggression model was overall significant compared to the null model lacking period (*X* ^*2*^ = 11.94, *df* = 3, *p* = 0.01). The interactions between period and receiver type (*X* ^*2*^ = 1.07, *df* = 1, *p* = 0.30), and giver and receiver type (*X* ^*2*^ = 0.13, *df* = 1, *p* = 0.71) were nonsignificant, therefore, results here represent a reduced model lacking both terms. After births, mothers increased their aggression more than nonmothers (Figure 4), as the interaction between period and giver‐type was significant (Table 6). In addition, nonmothers received generally more aggression than mothers, independent of period and giver‐type (Table 6).

**TABLE 6:** Results of the reduced aggression model (including estimates, standard errors, confidence levels and significance tests).

Term	Estimate	SE	*CL*_lower_	*CL*_upper_	*X* ^*2*^	*df*	*p*
Intercept	−7.494	1.341	−13.505	−5.757			
Period†	3.071	1.122	1.650	7.666			
Giver‐type‡	1.741	1.136	0.129	6.340			
Receiver‐type‡	2.073	0.840	0.859	5.599	7.315	1	**0.007**
Group size§	−0.099	0.258	−0.624	0.418	0.147	1	0.701
Period:giver‐type	−2.438	1.149	−6.834	−0.689	6.547	1	**0.011**

^†^ Dummy coded with before being the reference level.

^‡^ Dummy coded with mother being the reference level.

^§^ z‐transformed; mean and sd of original group size were 8.827 and 1.719, respectively.

## 5 Discussion

In this study, we examined patterns of infant handling and changes in social interactions after infant births in redfronted lemurs. Infant handling mostly consisted of infant grooming, whereas allomaternal care behaviors and aggressive infant handling were only observed very rarely. Infants were attractive to most group members, as individuals of all age‐sex classes handled infants at similar rates with a trend of slightly higher infant grooming rates in juvenile females. The presence of infants did not influence grooming interaction rates, but mothers received more approaches after giving birth. Proximity patterns also changed with mothers being closer to both mothers and nonmothers when they had infants, while the proximity between nonmothers did not change due to the presence of infants. Finally, we found an increase in aggression after births, which was particularly pronounced in mothers. Overall, these analyses indicate that the presence of infants leads to changes in the social interactions of mothers and can thereby even cause changes in overall group cohesion.

### 5.1 Infant Handling

In this study, we first report the occurrence of allomaternal care, in the form of holding or carrying infants, in redfronted lemurs. These behaviors only occurred in rare circumstances but could last several minutes. All cases of carrying were either between closely related adult females (mother‐daughter dyads) or by adult males, who were potential fathers. Notably, in all cases where infant transfer was observed, the infant rather than the mother or handler initiated the transfer. Overall, the low rates of allomaternal care and the increased rates of maternal aggression suggest that maternal tolerance of infant handling is low. However, the low rates of allomaternal care observed in this study may be partly due to the short study period, as allomaternal care in Eulemurs may become slightly more frequent later during an infant's development (Volampeno et al. 2011).

There were only four instances of negative infant handling observed, although female‐female competition is strong in redfronted lemurs, even resulting in evictions (Kappeler and Fichtel 2012; Prox et al. 2023) and infanticide (Jolly et al. 2000). This rare occurrence of negative infant handling supports earlier descriptions of low rates of aggression towards infants in this species (Barthold et al. 2009). Additionally, it also reflects the generally low aggression rates in this species (Ostner and Kappeler 1999).

Redfronted lemurs mostly performed affiliative/neutral infant handling, in particular in the form of grooming. Infant grooming was mostly initiated by handlers, but as infants became more active, they also sometimes initiated infant grooming interactions. Individuals of all age‐sex classes performed infant grooming at similar rates, although there was an indication that juvenile females groomed infants at slightly higher rates. However, these higher infant handling rates in juvenile females were predominantly driven by two mother‐daughter dyads, which resembles findings from other species where siblings display disproportionately high rates of infant handling (Nicolson 1987). In contrast, the lack of difference in infant grooming rates between adult females and males differs from observed infant handling patterns in most Cercopithecoidea, where males usually display lower rates of infant handling (Dunayer and Berman 2018; Meredith 2015) with few exceptions (Small 1990; Whitten 1987). Nonetheless, our results resemble earlier findings in other lemur species (Tecot and Baden 2018; Volampeno et al. 2011). The lack of sex difference in this study generally indicates that male redfronted lemurs are also attracted to infants.

Twinning did not influence infant grooming rates, which somewhat contrasts reports from other species, where twins were handled more often (Bennett 1988; Dielentheis et al. 1991; Kishimoto et al. 2014). Similarly, Manson (1999) reported higher approach rates to mothers in one case of twins compared to two single infants in white‐faced capuchins (*Cebus capucinus*). However, counting twins as one infant did not influence infant handling results in Silk (1999) study, suggesting that there was likely little difference in the infant handling received by twin bonnet macaques (*Macaca radiata*) and that, similar to our findings, each individual twin received only about half the amount of handling compared to singleton infants. Previous reports of increased infant handling towards twins were mostly based on increases in allomaternal care behaviors (Bennett 1988; Dielentheis et al. 1991; Kishimoto et al. 2014). This suggests that in contrast to other species, redfronted lemur mothers are able to care for twins successfully without help from other group members at least until the age of 1 month. However, allomaternal care of twins may also become more frequent as infants age and become larger.

Overall, the pattern of infant handling behaviors in the first month of life of redfronted lemurs resembles those of most macaques and baboons, as allomaternal care behaviors are very rare and the majority of infant handling consists of affiliative/neutral interactions (Maestripieri 1994a; Ross and MacLarnon 2000). This trend agrees with the notion of Maestripieri (1994a), that allomaternal care is performed at lower frequencies in species with strong female‐female competition due to low maternal tolerance. However, one main difference seems to exist in the identity of handlers, since in most macaques and baboons females generally handle infants at higher rates than males (Dunayer and Berman 2018; Maestripieri 1994a).

In regard to group‐living lemurs, the proportion of male infant handling varies between species, with males handling infants frequently in pair‐living species (Curtis and Zaramody 1999; Tecot and Baden 2018) as well as blue‐eyed black lemurs (Volampeno et al. 2011), whereas infant handling in ring‐tailed lemurs is more often performed by females (Gould 1992; Nakamichi and Koyama 2000). While it is generally more common for male care to occur in pair‐living species (Lukas and Clutton‐Brock 2012), as fathers can directly increase their fitness by caring for their infant, it remains unclear why male infant handling is common in redfronted lemurs and blue‐eyed black lemurs, which appear to have a polygynandrous mating system. The slightly higher infant handling rates in juvenile females may indicate that to some degree infant handling in redfronted lemurs serves to gain maternal experience (Lancaster 1971). Compared to females, it seems less clear why male redfronted lemurs also exhibit relatively high infant handling rates. While it has been described that adult males associate with mothers in other primates, these males usually only handle infants rarely (Huchard et al. 2010). Infant grooming by males may serve to build a relationship with the infant or strengthen the existing relationship with its mother (Dunayer and Berman 2017, 2018; Nakamichi and Koyama 2000). In addition, infant handling could increase future mating success (Ménard et al. 2001; Whitten 1987), but such an effect has rarely been described. Proximately, variation in male infant handling may be due to variation in the time males spend around mothers and infants, which somehow modifies their physiology, which in turn modifies their behavior (Hrdy 2024). Different species (or different individuals within species) might just be at different points in the development of these relationships, but we currently do not possess the empirical data to begin testing this interesting hypothesis in redfronted lemurs. Therefore, the function and mechanisms of male infant grooming need to be investigated in more detail in the future.

### 5.2 Changes in Affiliative and Affinitive Relationships

We did not find any indication of changes in grooming rates after the birth of infants, which does not align with our first prediction (Ia). This result is in contrast to findings in a variety of other species, where mothers receive more grooming after giving birth (Fruteau et al. 2011; Tiddi et al. 2010; Wei et al. 2013). Such changes are often related to infant handling, as mothers exchange infant access for grooming in biological markets (Henzi and Barrett 2002). Even though redfronted lemurs generally exchange grooming according to market rules (Port et al. 2009) and handle infants relatively frequently, there was no increase in grooming towards mothers. This may indicate that infant access is not considered a commodity in biological markets of redfronted lemurs, and that grooming is not necessary to access infants. However, in some previous studies, the occurrence of grooming rather than its duration positively affected infant handling rates (Gumert 2007; Tiddi et al. 2010). Additionally, grooming rates towards mothers may have been slightly underestimated, as grooming a mother's ventrum was considered infant handling, when it was impossible to distinguish whether the grooming was directed at the infant or the mother. Studying grooming exchanges in the context of infant handling more closely in the future is thus necessary to provide more insight into the role of infants in this species and their potential value in biological markets.

While we did not find changes in grooming interaction rates for any dyad types, mothers were clearly attractive, as they received more approaches after giving birth, which supports prediction Ib. Increased approaches towards females with dependent young have been observed in many different primate species (e.g., Altmann 1980; Martel et al. 1994; Slater et al. 2007; Wei et al. 2013) and may indicate attraction to their infant and an intent to interact with it, similar to an increase in the time spent in close proximity (Dunayer and Berman 2017). The increase in approaches may result in stronger relationships with mothers if individuals also stay in close proximity to them, thereby the presence of infants may be an initiator of the strengthening of social relationships for females (Dunayer and Berman 2018).

While mothers received more approaches after infants were born, in contrast to prediction II, the approach rates within nonmother‐nonmother dyads did not change. This suggests that there is no trade‐off for nonmothers between being close to mothers or other group members. Thus, nonmothers may generally spend more time in close proximity to other group members during the birth season. This finding contrasts with those of an earlier study in golden snub‐nosed monkeys, where approach rates between nonmother‐nonmother dyads decreased after births (Wei et al. 2013). This observation may indicate that redfronted lemurs have a relatively relaxed time budget and can increase their time spent being social in specific situations (Lehmann et al. 2007). Time constraints are, however, likely more pronounced for other social behaviors, such as grooming, rather than proximity seeking. Additionally, due to their cathemerality, changes in time budget could be balanced out throughout the night or their overall active time may vary.

Similar changes also applied to overall proximity, as there was no change in spatial distance between nonmothers, but individuals were closer after birth in both mother‐mother and mother‐nonmother dyads. This contradicts our third prediction that spatial cohesion would decrease as mothers may avoid other group members, which was based on an earlier description of maternal behavior of redfronted lemurs, where mothers were found to distance themselves from other group members except their yearling offspring (Overdorff 1998). In contrast, mothers in our study likely did not perceive other group members, especially adult males, as a major threat to their infants. Instead, the increase in proximity once again highlights the attractiveness of mothers to other group members and suggests that mothers were not only approached more but that the dyads stayed more in proximity as well. While an increase in spatial cohesion could also be the result of ecological changes during the dry season, such as the growing limitation of water availability or increasing temperatures, this possibility seems unlikely, as it should also have resulted in a change in spatial distance between nonmothers. Furthermore, these seasonal climatic changes also did not affect male social behavior in the same population (Prox et al. 2025).

Mothers not only associated more with nonmothers but also with other mothers, as they are closer together after birth than before. Associations between mothers have previously been described in other species (Maestripieri 1994b; Mielke et al. 2020; Ostner and Schülke 2018) and are assumed to potentially benefit infants by providing them with playing partners (Ostner and Schülke 2018; Wasser 1983). However, since there were few mother‐mother dyads in general, the increase in proximity between mothers was mostly driven by three females from one group. In contrast, other mother‐mother dyads spent barely any time together. Therefore, associations between mothers seem to be highly differentiated, but it remains unclear what drives these associations or the lack thereof. While most associations between mothers in our study involved closely related females (i.e., mother‐daughter or sibling dyads), not all closely related mothers associated with each other. Therefore, associations between mothers may additionally depend on multiple factors including kinship, but also the number of mothers per group, as well as the general group size and composition.

## 6 Changes in Aggression

In addition to changes in affinitive behaviors, we found an increase in aggression, which was especially pronounced in mothers, supporting prediction IV. Maternal aggression directed at other group members is a common mechanism to protect infants in many species (Maestripieri 1992), including other lemurs (Fornasieri and Roeder 1992; Sauther 1993). Depending on the species, such aggression can be mainly directed at strange males (or non‐fathers more generally; Pereira and Weiss 1991) or at individuals attempting to handle infants (Maestripieri 1994c; Troisi 1988). Alternatively, maternal aggression could arise from an increase in female‐female competition during the birth season (Baniel et al. 2018; Maestripieri 1994a). In this study, the majority of maternal aggression was directed at nonmothers, in particular juveniles (83 of 97 aggression events) and seemed to mostly occur during infant handling attempts rather than the seeking of maternal attention. This suggests that maternal aggression in redfronted lemurs served mostly to prevent infant handling, rather than being the result of female competition or infanticide prevention.

While mothers exhibited a stronger increase in aggression, aggression rates also increased in nonmothers. The aim behind this aggression may be the protection of infants, as a large number of aggression events between nonmothers were initiated by adult males while in close proximity to mothers (41 of 94 aggression events). Protection of infants by males through aggression has been described in multiple primate species (e.g. Altmann 1980; Fruteau et al. 2010) and is considered to be one of the most common forms of male care in primates and to have the biggest impact on an infant's fitness (Hrdy 1976; Rosenbaum and Silk 2022; Van Schaik and Kappeler 1997).

## 7 Implications

The bonds between males and mothers exhibited a particularly noticeable shift in social relationships, which may be connected to direct fitness benefits. Unlike what has been described previously in other seasons (Ostner and Kappeler 1999), there does not seem to be one central male after infant births with respect to proximity to females and infant handling. In contrast, while some males associated with multiple mothers, some mothers were also associated with multiple males (Figure S5). This may suggest that central males cannot monopolize multiple females during the birth season, especially when mothers do not associate with each other. Instead, central males may have to decide which female to associate with, but it is unclear what their decision is based on. Paternity likelihood alone is unlikely to explain their decision, as central males typically have the highest mating success with all females in their group (Ostner and Kappeler 1999) and the highest reproductive success (Kappeler and Port 2008).

Overall, the associations between mothers and males and the increase in maternal aggression indicate that the social environment likely exposes infants to risk, even though barely any direct aggression was directed at infants. Infanticide by both males and females has been previously described in redfronted lemurs (Jolly et al. 2000). In addition, the natal coat, which resembles males, has been presumed to reduce female‐female competition and female infanticide (Barthold et al. 2009). Therefore, social relationships in the birth season, especially the associations between males and mothers, may be crucial for infant survival. About 27% of redfronted lemur infants do not survive until 4 months of age (Ostner and Kappeler 2004) and infant survival has previously been found to be negatively associated with the number of juvenile females in the group (Prox et al. 2023). This may be due to indirect competition (Kerhoas et al. 2014) or a reduced likelihood of successfully rearing offspring multiple times in a row (Lee et al. 2019; Overdorff et al. 1999). However, infant deaths could also be an indirect result of rough infant handling by juveniles (Kleindorfer and Wasser 2004), who are yet to display proficient maternal skills (Meaney et al. 1990), as juvenile females expressed the highest rates of infant handling, but also received much maternal aggression.

This study was one of the first studies to compare changes in social relationships of the whole group after infant births. By including affiliative, affinitive and aggressive behaviors, we were able to examine changes in different aspects of social relationships. In addition, combining changes in social interactions with infant handling enabled us to highlight the social effects of infant handling. Due to the unexpected birth of multiple pairs of twins within our study groups, this was one of the first studies comparing infant handling behaviors between multiple singleton and twin births in a normally monotocous species. However, as the exact timing of births could not be planned, observational timeframes before and after birth varied slightly between groups. Changes in social relationships likely reduce with increasing infant age (Giraud et al. 2021; Nakamichi and Koyama 2000), but, in ring‐tailed lemurs, contact rates of mothers remained elevated until their infants were 9 weeks old (Nakamichi and Koyama 2000), which corresponds to the oldest age of infants in this study. Therefore, while changes in relationships may have been more pronounced if analyses had been strictly restricted to the first month after infant births, different results would not necessarily have been expected. Additionally, as changes in social relationships may already occur during pregnancy or in anticipation of births (e.g., Karaskiewicz et al. 2021), it would be necessary to include data from longer periods before birth to obtain a more comprehensive comparison of the social relationships after birth compared with nonreproductive season social behavior. By combining social interaction rates in the birth season with infant development measures and data on infant survival, future studies may determine how these social relationship patterns may impact infants. Also, to determine whether male infant grooming is related to paternity or may function to increase future mating success, mating data from the previous and subsequent season as well as paternity data should be considered.

## 8 Conclusions

This study illustrated that redfronted lemur infants are attractive to other group members. However, their infant handling pattern is relatively unique compared to most Cercopithecoidea, as allomaternal care behaviors occurred only rarely, but males performed comparatively high rates of infant handling. Even though redfronted lemurs express strong female competition, infants were rarely the target of harassment, which suggests that there are likely some mechanisms protecting infants from overt aggression, such as associations between mothers and males. Overall, male redfronted lemurs seem to play an important role in the early life of infants and intersexual relationships may thus be crucial for their fitness. In addition, this study highlights that the interaction patterns of mothers differ due to the presence of newborn infants, with further effects on overall social structure.

## Author Contributions

**Amrei Pfaff:** conceptualization (equal), formal analysis (equal), investigation (equal), methodology (equal), writing – original draft (equal). **Claudia Fichtel:** conceptualization (equal), supervision (equal), writing – review and editing (equal). **Peter M. Kappeler:** conceptualization (equal), supervision (equal), writing – review and editing (equal).

## Conflicts of Interest

The authors declare no conflicts of interest.

## Supporting information


**Figure S1:** Stability of reduced approach model estimates.
**Figure S2:** Stability of reduced aggression model estimates.
**Figure S3:** Stability of grooming model estimates.
**Figure S4:** Stability of infant handling model estimates.
**Figure S5:** Social networks based on proximity scores of four groups before and after the birth of infants.
**Table S1:** Group compositions of four study groups.

## Data Availability

All data and code used in this study are available on figshare at https://doi.org/10.6084/m9.figshare.28070381.
