# Supplementary material for: Infants as Social Magnets: The Influence of Births on Social Interactions in Redfronted Lemurs (Eulemur rufifrons)
Source: Am J Primatol. 2025 Aug 18;87(8):e70067. doi: 10.1002/ajp.70067 (PMC12360686; doi:10.1002/ajp.70067)
Supplement: Supplementary file 1 — Figure S1: Stability of reduced approach model estimates. Figure S2: Stability of reduced aggression model estimates. Figure S3: Stability of grooming model estimates. Figure S4: Stability of infant handling model estimates. Figure S5: Social networks based on proximity scores of four groups before and after the birth of infants. Table S1: Group compositions of four study groups. [file AJP-87-e70067-s001.docx]

**Supporting Information for Infants as social magnets: The influence of births on social interactions in redfronted lemurs (*Eulemur rufifrons*) by Amrei Pfaff, Claudia Fichtel and Peter M. Kappeler**

**Additional Information on Model Fitting process**

In the grooming, approach and aggression models, we initially included all theoretically identifiable random slopes, namely the three-way-interaction of period*giver-type*receiver-type within group identity and the interaction between period*giver-type within receiver identity and the interaction period*receiver-type within giver identity. We further included all correlations between random intercepts and slopes into all models initially (Barr et al., 2013). In the aggression model, a “singular fit” message suggested that some random effect terms were unidentifiable. Therefore, we excluded the correlation parameters within the random factor group identity. This decision led to only a small decrease in model fit (log-likelihoods: with correlation parameters: -355.92, *df* = 57; without correlation parameters: -356.09, *df* = 36). The initial models for grooming and approaches did not converge. Therefore, we started excluding correlations between random intercepts which were essentially one, suggesting that they were not identifiable (Matuschek et al., 2017). The final approach model did not include any correlations. As convergence issues remained after excluding all correlation parameters from the grooming model, we started dropping random slopes that were estimated to be essentially zero. The resulting grooming model did not include any random slopes within group identity while maintaining the original random slopes within giver and receiver identity.

**Table S1:** Group compositions of four study groups.

| **Group** | **Adult females** | **Adult males** | **Juvenile females** | **Juvenile males** | **Group size** |
| --- | --- | --- | --- | --- | --- |
| alpha | 4 | 4/3 | 2 | 1 | 11/10 |
| B | 2 | 3 | 1 | 2 | 8 |
| J | 2 | 4 | - | 2 | 8 |
| S | 1 | 2 | 1 | - | 4 |

**Table S2:** Overview of infants born during the study period.

| **Infant** | **Mother** | **Group** | **Twinning** | **Birth date** | **Disappearance date** |
| --- | --- | --- | --- | --- | --- |
| Rbdbb | Rbd | alpha | Singleton | 2023-10-21 | - |
| Genbb | Gen | alpha | Singleton | 2023-10-21 | - |
| Redbb | Red | alpha | Twins | 2023-11-16 | - |
| Flobb | Flo | alpha | Singleton | 2023-11-18 | - |
| Sapbb | Sap | B | Singleton | 2023-10-05 | 2023-11-01 |
| Isabb | Isa | B | Singleton | 2023-11-01 | 2023-11-19 |
| Palbb | Pal | J | Twins | 2023-09-27 | - |
| Cambb | Cam | J | Singleton | 2023-10-03 | - |
| Rabbb | Rab | S | Twins | 2023-10-22 | - |

**
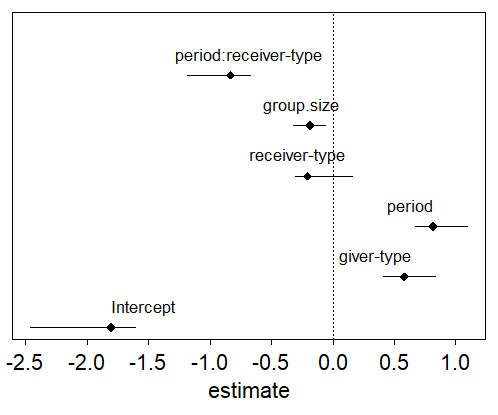
**

**Figure S1:** Stability of reduced approach model estimates. Model stability is calculated by excluding levels of random effects one at a time. Diamonds indicate actual model estimates and lines represent range of estimates from fitted models with excluded random effect levels. Stability was visually assessed, with good stability being indicated by little variation around the estimates.

**
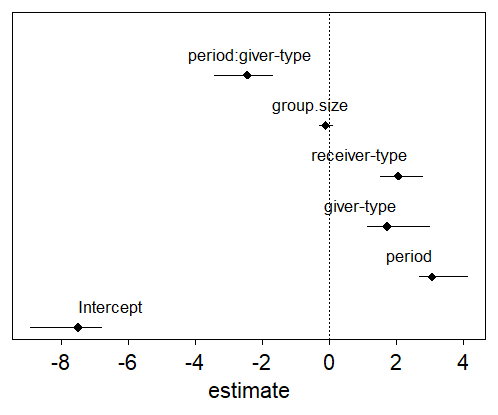
Figure S2:** Stability of reduced aggression model estimates. Model stability is calculated by excluding levels of random effects one at a time. Diamonds indicate actual model estimates and lines represent range of estimates from fitted models with excluded random effect levels.

**
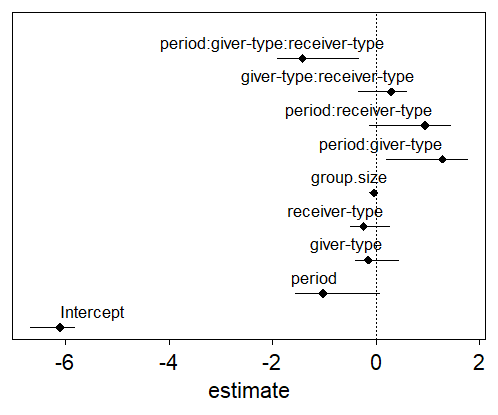
**

**Figure S3:** Stability of grooming model estimates. Model stability is calculated by excluding levels of random effects one at a time. Diamonds indicate actual model estimates and lines represent range of estimates from fitted models with excluded random effect levels.

**
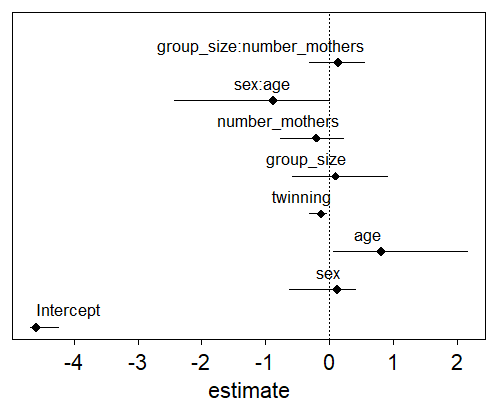
Figure S4:** Stability of infant handling model estimates. Model stability is calculated by excluding levels of random effects one at a time. Diamonds indicate actual model estimates and lines represent range of estimates from fitted models with excluded random effect levels.


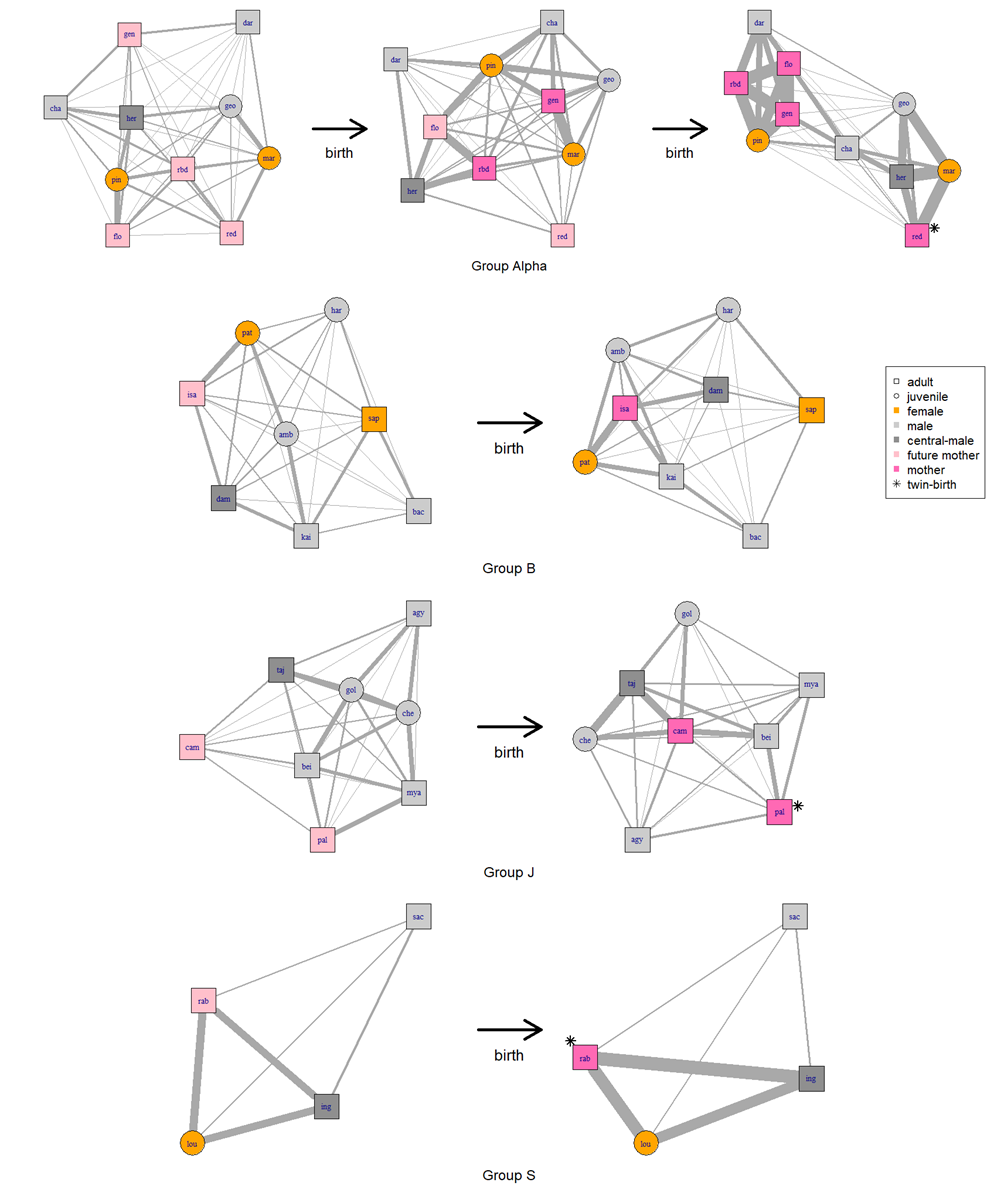


**Figure S5:** Social networks based on proximity scores of four groups before and after the birth of infants. The line width represents the proximity score of the dyad. Circles represent juveniles and squares represent adults. Pink squares represent mothers before (light pink) and after (dark pink) giving birth, orange depicts nonmother females and grey depicts males, with dark grey depicting the central male of each group. Asterisks depicts the occurrence of a twin birth.
